# Health related quality of life in anti interferon γ autoantibody associated immunodeficiency syndrome measured with EQ5D5L and SF36

**DOI:** 10.1038/s41598-023-41340-w

**Published:** 2023-09-01

**Authors:** Jirat Temsangsukmanee, Wannada Laisuan, Kunlawat Thadanipon, Prapaporn Pisitkun, Pintip Ngamjanyaporn, Thanitta Suangtamai, Supa Oncham, Prawat Chantharit, Porpon Rotjanapan

**Affiliations:** 1 Faculty of Medicine, Ramathibodi Hospital, Mahidol University, Bangkok, Thailand; 2 Division of Allergy Immunology and Rheumatology, Department of Medicine, Faculty of Medicine Ramathibodi Hospital, Mahidol University, 270 Rama VI Road, Rajataewe, Bangkok, 10400 Thailand; 3 Division of Dermatology, Department of Clinical Epidemiology and Biostatistics, Department of Medicine, Faculty of Medicine, Ramathibodi Hospital, Mahidol University, Bangkok, Thailand; 4 Division of Infectious Diseases, Department of Medicine, Faculty of Medicine Ramathibodi Hospital, Mahidol University, Bangkok, Thailand

**Keywords:** Human behaviour, Immunological deficiency syndromes, Medical research

## Abstract

The anti-IFN-γ disease is a rare condition characterized by recurrent and persistent infections, potentially impacting the quality of life (QoL). However, comprehensive data on QoL in this population are lacking. This study aims to evaluate the QoL of Anti-IFN-γ patients compared to healthy control and explore potential differences in QoL between patients in the active and remission stages. A cross-sectional study design was conducted, recruiting 38 Anti-IFN-γ patients and 38 sex- and age-matched healthy controls. QoL assessment utilized the 5-level EuroQol-5 Dimension (EQ-5D-5L) and the 36-Item Short Form Health Survey (SF-36). The Anti-IFN-γ group had a mean age of 57.37 (± 10.32) years, with females comprising 60.53%. Among the Anti-IFN-γ patients, 55.26% were classified as having active disease. 63% of Anti-IFN-γ patients received Immunosuppressive treatments. Anti-IFN-γ disease exhibited a significant negative impact on HRQoL, as evidenced by lower utility scores in EQ-5D-5L and lower physical and mental component scores in SF-36 across various domains, including physical function, role physical, general health, bodily pain, social functioning, role emotion and mental health, compared to healthy controls. Additionally, patients in the active disease displayed lower scores in multiple domains, including bodily pain, general health, role emotion and mental health, and a lower utility score in EQ-5D-5L compared to patients in remission. The anti-IFN-γ disease significantly impairs the HRQoL of affected individuals compared to healthy controls. However, effective treatment leading to remission holds promise for improving the HRQoL of patients with Anti-IFN-γ disease.

## Introduction

Anti-interferon-γ autoantibodies (Anti-IFN-γ) is a rare disease with a prevalence of 0.5–1 per million in general populations^1^. Over the last decade, more than 600 reported cases have emerged, primarily from Southeast Asia, particularly Thailand and Taiwan^2–5^. This condition typically manifests in adulthood around the age of 50 years^2,6,7^. Multiple opportunistic infections affecting various organs have been documented, including nontuberculous mycobacterium (NTM) infections observed in 85.5% of Anti-IFN-γ patients and salmonellosis in 18.8%. In addition, viral and fungal infections have been reported^1,2,5^. The organs most affected by these infections include lymph nodes, bone, lungs, and skin^1,5^.

Anti-IFN-γ patients often experienced a chronic clinical course characterized by recurrent and persistent infections, with reported rates ranging from 13 to 75%. The mortality rate associated with this condition varies between 0 and 24%^1,8,9^. In a case series from Taiwan by Chi et al.^9^, sixty percent of patients had persistent NTM infections requiring long-term antimicrobial therapy. The median time to recurrence was 2.5 episodes, with recurrence infections documented after discontinuing antimicrobial treatment for 1–2 months. Hase et al.^5^ reported that 75% (83/99 patients) of Anti-IFN-γ patients experienced recurrence or relapse infections after discontinuing anti-NTM treatments for one month to 2.5 years. Only 12% (13/99) of patients achieved remission, defined as being free from active disease and showing a complete response. These findings suggest that Anti-IFN-γ diseases are associated with prolonged recurrent and persistent infections, which can significantly impact the quality of life. However, limited quality of life data is available to guide clinicians in managing these conditions.

## Methods

### Study design and participants

The study was approved by the Institutional Review Board of Ramathibodi Hospital (MURA2021/152) and was granted by Mahidol University. Written informed consent was obtained from all patient. The study was conducted in accordance with the International Conference on Harmonisation-Good Clinical Practice (ICH-GCP) guidelines. A cross-sectional design study was performed at Ramathibodi Hospital, a tertiary care center. The committee approved the use of patient data for the publication in this study.

Thirty-eight patients diagnosed with Anti-IFN-γ associated immunodeficiency syndrome were enrolled from a prospective registry database and classified into two groups based on the disease activity. The diagnosis of Anti-IFN-γ disease included the positive finding of Anti-IFN-γ autoantibodies through inhibitory ELISA^10^, as well as the absence of human immunodeficiency viral infection. Remission was defined as controlled infection and meeting all criteria, which included (1) the recovery of all the symptoms reported before therapy, (2) resolution of previously positive findings on physical examination and (3) normal biomarkers such as C-reactive protein, white blood cell count, and interleukin-6^6^. Active disease referred to Anti-IFN-γ patients who did not meet all criteria stated. A group of 38 healthy controls with no documented underlying diseases, matched for sex and age, were also enrolled for comparison.

The participants completed self-administered quality of life questionnaires, including the 5-level EuroQol-5 Dimension (EQ-5D-5L) and 36-Item Short Form Health Survey (SF-36).

### Measures

The quality of life (QoL) was assessed using the 5-level EuroQol-5 Dimension (EQ-5D-5L) and 36-Item Short Form Health Survey (SF-36). The Thai version of the SF-36 and EQ-5D-5L were validated and utilized^11,12^.

The EQ-5D-5L questionnaire assessed the quality of life based on five dimensions: mobility, self-care, usual activities, pain/discomfort, and anxiety/depression. Each dimension has five levels ranging from no problems to extreme problems (Level 1 = no problems, Level 2 = slight problems, Level 3 = moderate problems, Level 4 = severe problems and Level 5 = extreme problems). The EQ-5D-5L responses were converted into a utility score reflecting an individual’s health status, ranging from − 1 to 1. A higher score indicates a better health status.

The SF-36 survey consists of 36 items divided into eight domains: physical functioning (PF), role physical (RP), role emotion (RE), social functioning (SF), mental health (MH), vitality (VT), bodily pain (BP) and general health (GH). The final scores for each domain were calculated, where lower scores indicated poorer health-related quality of life (HRQoL).

### Statistical analysis

Statistics were performed using the Stata version 17.0 (StataCorp. 2021. College Station, TX.). Continuous variables were expressed as mean and standard deviation. Categorical variables were expressed as numbers and percentages. Two sample t-tests were used to determine the difference between Anti-IFN-γ and healthy controls. *P* values < 0.05 were considered statistically significant.

## Results

### Health-related quality of life (HR-QoL) compares anti-IFN-γ patients and healthy controls

#### Demographic characteristics

Table 1 shows the demographic characteristics of the participants. Thirty-eight Anti-IFN-γ patients, with a mean age of 57.37 years (± 10.32), were recruited, with females comprising 60.53% (23/38 patients). The median duration of IFN- γ Auto Ab disease from the onset of the disease to the enrollment date was 5.34 (2.42–6.90) years. Anti-IFN-γ patients were classified into two groups according to the criteria, active disease was defined in 21 out of 38 patients (55.26%), and remission was observed in 17 out of 38 patients (44.74%). Sixty-three percent of patients in the active and remission stage received immunosuppressive treatment, including azathioprine in 34.21% (13/38 patients), Rituximab in 18.42% (7/38 patients), Cyclophosphamide in 7.89% (3/38 patients) and prednisolone 2.63% (1/38 patients). Thirty-eight healthy controls, matched for sex and age, were enrolled, with a mean age of 56.66 (± 4.99) years.

**Table 1:** Demographic characteristics.

Characteristics	IFN- γ Auto Ab (n = 38)	Healthy (n = 38)	*p* value
Sex, n (%)			0.5000
Male	15, (39.47)	15 (39.47)	
Female	23, (60.53)	23 (60.53)	
Age, mean ± SD years	57.37 (10.32)	56.66 (4.99)	0.7037
Duration of IFN- γ Auto Ab disease, median (IQR) years	5.34 (2.42–6.90)	0	
Disease activity			
Active	21, (55.26)	0	
Remission	17, (44.74)	0	
Current medical treatment of IFN- γ Auto Ab			
Immunosuppressive drugs, n (%)			
Azathioprine	13, (34.21)	0	
Rituximab	7, (18.42)	0	
Cyclophosphamide	3, (7.89)	0	
Prednisolone	1, (2.63)	0	
Antimicrobial agents	7, (18.42)	0	
No medication	8, (21.05)	38 (100)	

### EQ-5D-5L

**Figure 1:**
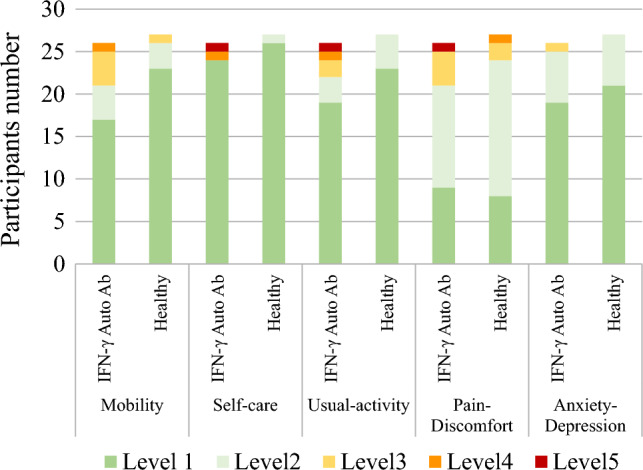
EQ-5D-5L compare between Anti-IFN-γ autoantibodies patients and healthy controls.

The EQ-5D-5L data was converted into a utility score, as presented in Table 2 and Fig. 1. The utility score, also known as the EQ-5D-5L index, was lower in the Anti-IFN-γ group, with a mean score of 0.86 (± 0.17), compared to 0.95 (± 0.06) in the healthy control group. This difference is statistical significance (*p* < 0.0025). Furthermore, more Anti-IFN-γ patients experienced moderate to extreme problems (level 3–5) in the mobility, self-care, usual activities, pain/discomfort and anxiety/depression domains compared to the healthy controls.

**Table 2:** SF-36 and EQ5D-5L in Anti-IFN-γ autoantibodies patients compare with healthy controls.

	IFN-γ Auto Ab (n = 38)	Healthy (n = 38)	*p* value
Health status variables, mean (± SD)			
SF-36 domains			
Physical function	23.45 (0.87)	27.03 (2.47)	0.0004*
Role physical	5.75 (1.59)	7.37 (1.28)	0.0000*
Bodily pain	3.71 (0.22)	4.32 (0.87)	0.0220*
General health	15.02 (5.14)	19.45 (2.88)	0.0000*
Vitality	16.32 (2.28)	16.45 (1.89)	0.7853
Social function	7.79 (2.20)	9.13 (1.14)	0.0013*
Role emotional	4.79 (1.36)	5.66 (0.63)	0.0006*
Mental health	20.76 (3.82)	23.00 (1.58)	0.0013*
Report health transition	2.34 (1.40)	2.71 (0.93)	0.1804
EQ-5D-5L index	0.86 (0.17)	0.95 (0.06)	0.0025*

*p < 0.05.

### SF-36

**Figure 2:**
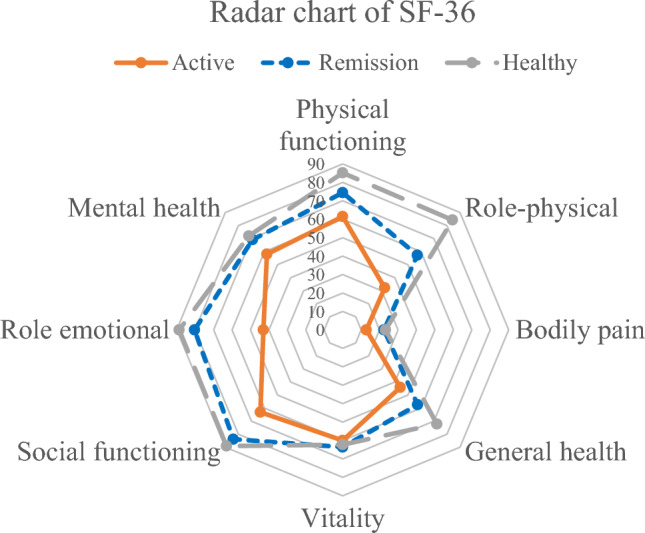
Radar chart of SF-36.

The SF-36 results in Table 1 and Fig. 2 reveal a significant difference in SF-36 domain scores between the Anti-IFN-γ group and the healthy controls, indicating lower physical and mental well-being in the Anti-IFN-γ group. The domains that demonstrated significant disparities include physical function (PF) (*p* = 0.0004), role physical (RP) (*p* = 0.0000), general health (GH) (*p* = 0.000), bodily pain (BP) (*p* = 0.0220), social functioning (SF) (*p* = 0.0013), role emotion (RE) (*p* = 0.0006) and mental health (MH) (*p* = 0.0013). However, no significant differences were observed in vitality (VT) (*p* = 0.7853) and report health transition (*p* = 0.1804).

Specifically, the mean scores for the physical components scores, including PF, RP and BP, were 23.45 (± 0.87), 5.75 (± 1.59) and 3.71 (± 0.22), respectively, in the Anti-IFN-γ group, in comparison to 27.03 (± 2.47), 7.37 (± 1.28) and 4.32 (± 0.87) in the healthy control group. Additionally, the mean scores for the mental components score, comprising SF, RE and MH, were 7.79 (2.20), 4.79 (1.36) and 20.76 (3.82), respectively, in the Anti-IFN-γ group compared to 9.13 (1.14), 5.66 (0.63) and 23.00 (1.58) in the healthy control group.

### Subgroup analysis of health-related quality of life (HR-QoL) compares active and remission in anti-IFN-γ patients

#### EQ-5D-5L

The results in Table 3 demonstrate that Anti-IFN-γ patients in the active stage have a lower utility score, with a mean of 0.81 (± 0.20), compared to Anti-IFN-γ patients in remission, who have a mean score of 0.92 (± 0.09). The difference was reached statistically significant (*p* = 0.0305).

**Table 3:** Subgroup analysis of SF-36 and EQ5D-5L in active Anti-IFN-γ autoantibodies compare with remission.

	Active (n = 21)	Remission (n = 17)	*p* value
Health status variables, mean (± SD)			
SF-36 domains			
Physical function	22.29 (5.47)	24.88 (5.02)	0.1401
Role physical	5.29 (1.52)	6.29 (1.53)	0.0503
Bodily pain	3.28 (1.35)	4.24 (1.15)	0.0270*
General health	13.83 (4.97)	16.49 (5.11)	0.0184*
Vitality	16.00 (1.82)	16.71 (2.76)	0.3495
Social function	7.05 (2.33)	8.71 (1.65)	0.1131
Role emotional	4.29 (1.38)	5.41 (1.06)	0.0091*
Mental health	19.52 (3.66)	22.29 (3.55)	0.0242*
Report health transition	2.52 (1.57)	2.12 (1.52)	0.3813
EQ-5D-5L index	0.81 (0.20)	0.92 (0.09)	0.0305*

*p < 0.05.

#### SF-36

Table 3 illustrates the disparities of SF-36 domains between Anti-IFN-γ patients in the active and remission stages. Patients in the active stage exhibited lower scores in multiple domains, including BP, GH, RE and MH. Specifically, the mean scores in these domains were 3.28 (± 1.35), 13.83 (± 4.97), 4.29 (± 1.38) and 19.52 (± 3.66), respectively, compared to 4.24 (± 1.15), 16.49 (± 5.11), 5.41 (± 1.06) and 22.29 (+ 3.55) in remission group.

## Discussion

Anti-IFN-γ associated immunodeficiency syndrome is a rare disease^1,2^, characterized by recurrent or persistent infections, particularly NTM infections, which can reoccur after discontinuing antimicrobial treatment for a period of 1 month–2.5 years^5,9^. This condition commonly affects multiple organs, including lymph nodes, skin, lungs and bone, resulting in a significant impact on the quality of life. Although adjunctive immunosuppressive therapy has shown favorable clinical outcomes, approximately 20–30% of Anti-IFN-γ patients experienced partial remission or do not respond to treatment^6,13^. To the best of my knowledge, a limited amount of data is available on the quality of life and standard treatment for this condition. Therefore, this study represents the first report on the impact of HRQoL in Anti-IFN-γ patients compared to healthy controls.

The health-related quality of life (HRQoL) was assessed using the validated Thai versions of EQ-5D-5L and SF-36. Table 1 provides the demographic characteristics of Anti-IFN-γ patients and sex-and age-matched healthy controls. The mean age of the Anti-IFN-γ group was 57.37 (± 10.32) years, while the mean age of the healthy control was 56.66(± 4.99) years. The difference in mean age between the two groups did not reach statistically significant. The median duration of disease in the Anti-IFN-γ group was 5.34 (2.42–6.90) years. Disease activities were classified into two groups based on the criteria outlined in the methods section, with 21 patients (55.26%) classified as active and 17 patients (44.74%) classified as being in remission. Immunosuppressive treatments were prescribed to 24 patients (63.6%) in the active and remission groups.

Anti-IFN-γ disease negatively impacted HRQoL, as evidenced by lower utility scores in EQ-5D-5L and lower physical and mental component scores in SF-36 compared to healthy individuals. The SF-36 assessment revealed a disruption in normal social activities due to physical and emotional problems experienced by Anti-IFN-γ patients. Statistically significant differences were observed in the physical components scores (PF, RP and BP) and mental components scores (SF, RE and MH).

Subgroup analysis comparing the active and remission stages of Anti-IFN-γ patients further demonstrated lower HRQoL in the active disease group compared to the remission group. This finding suggests that the quality of life is significantly worse in Anti-IFN-γ patients, particularly those in the active disease stage. However, treatment modalities leading to remission of Anti-IFN-γ disease have been shown to improve HRQoL. These results highlight the importance of effective treatment strategies in managing the disease and enhancing the well-being of Anti-IFN-γ patients.

The impairment of HRQoL in Anti-IFN-γ disease can be comparable to patients with diabetic mellitus and patients with human immunodeficiency viral infection (HIV). A previous systematic review using EQ-5D-5L in different diseases^14^ reported the health utility ranged from 0.31 to 0.99 for diabetic mellitus, with a meta-analysis random effect model of 0.83, (95% CI 0.77–0.90) and health utility ranged from 0.65 to 0.90 in HIV infection, with meta-analysis random effect model: 0.84, (95% CI 0.80–0.88). In this study, Anti-IFN-γ disease had a utility range of 0.31 to 1.00, with a mean utility score of 0.86 (± 0.17). In subgroup analysis, the active Anti-IFN-γ group showed a health utility range of 0.311 to 1, with a mean utility score of 0.81(± 0.20), while the remission Anti-IFN-γ group had a health utility range of 0.635 to 1, with a mean utility score of 0.92 (± 0.09). These comparative results suggest that Anti-IFN-γ disease has a similar HRQoL as chronic diseases such as diabetic mellitus and HIV infection. However, the remission stage of Anti-IFN-γ disease exhibited better HRQoL.

This study has certain limitations that should be acknowledged. Firstly, the small number of participants results from the disease’s rarity, which may limit the generalizability of the findings. The cross-sectional design also has limitations, as it cannot establish causal relationships or demonstrate the long-term effects of treatment on HRQoL within the same Anti-IFN-γ patients. Additionally, this study did not directly compare HRQoL with other chronic diseases, which could provide further insights into the relative impact of Anti-IFN-γ disease on quality of life. These limitations highlight the need for larger longitudinal studies and comparative analyses to gain a more comprehensive understanding of the HRQoL implications in Anti-IFN-γ patients and to compare their experiences with those of individuals with other chronic diseases.

## Conclusion

Anti-IFN-γ associated immunodeficiency syndrome was found to have a detrimental effect on HRQoL, as evidenced by lower utility scores in EQ-5D-5L and lower physical and mental compartment scores in SF-36 when compared to healthy controls. However, effective treatment leading to the remission stage has shown potential for improving HRQoL in patients with Anti-IFN-γ disease.

## Data Availability

The datasets generated and/or analysed during the current study are not publicly available due to ongoing cohort study but are available from the corresponding author on reasonable request.
